# Rationale for a home dialysis virtual ward: design and implementation

**DOI:** 10.1186/1471-2369-15-33

**Published:** 2014-02-14

**Authors:** Michael E Schachter, Joanne M Bargman, Michael Copland, Michelle Hladunewich, Karthik K Tennankore, Adeera Levin, Matthew Oliver, Robert P Pauly, Jeffrey Perl, Deborah Zimmerman, Christopher T Chan

**Affiliations:** 1 Department of Medicine, Division of Nephrology, University Health Network, 200 Elizabeth Street, Toronto, ON M5G 2C4, Canada; 2 Department of Medicine, Division of Nephrology, QEII Health Sciences Centre, 5820 University Avenue, Halifax, NS B3H 1V7, Canada; 3 Department of Medicine, Division of Nephrology, Vancouver General Hospital, University of British Columbia, 855 12th Avenue W, Vancouver, BC V5Z 1M9, Canada; 4 Department of Medicine, Division of Nephrology, St Paul’s Hospital, 1081 Burrard Street, Vancouver, BC V6Z 1Y6, Canada; 5 Department of Medicine, Division of Nephrology, Sunnybrook Health Sciences Centre, 2075 Bayview Avenue, Toronto, ON M4N 3M5, Canada; 6 Department of Medicine, Division of Nephrology & Transplantation Immunology, University of Alberta, 11-107 Clinical Sciences Building, 8440, 112th Street, Edmonton, AB T6G 2G3, Canada; 7 Department of Medicine, Division of Nephrology, St. Michael’s Hospital, 30 Bond Street, Toronto, ON M5B 1W8, Canada; 8 Department of Medicine, Division of Nephrology, Ottawa Hospital, University of Ottawa, 1967 Riverside Drive Ottawa, Ottawa, ON K1H 7W9, Canada

**Keywords:** Virtual ward, Home dialysis, Patient support, Hospital-avoidance, Peritoneal dialysis, Home hemodialysis, Re-admission, Technique survival, Patient satisfaction

## Abstract

**Background:**

Home-based renal replacement therapy (RRT) [peritoneal dialysis (PD) and home hemodialysis (HHD)] offers independent quality of life and clinical advantages compared to conventional in-center hemodialysis. However, follow-up may be less complete for home dialysis patients following a change in care settings such as post hospitalization. We aim to implement a Home Dialysis Virtual Ward (HDVW) strategy, which is targeted to minimize gaps of care.

**Methods/design:**

The HDVW Pilot Study will enroll consecutive PD and HHD patients who fulfilled any one of our inclusion criteria: 1. following discharge from hospital, 2. after interventional procedure(s), 3. prescription of anti-microbial agents, or 4. following completion of home dialysis training. Clinician-led telephone interviews are performed weekly for 2 weeks until VW discharge. Case-mix (modified Charlson Comorbidity Index), symptoms (the modified Edmonton Symptom Assessment Scale) and patient satisfaction are assessed serially. The number of VW interventions relating to eight pre-specified domains will be measured. Adverse events such as re-hospitalization and health-services utilization will be ascertained through telephone follow-up after discharge from the VW at 2, 4, 12 weeks. The VW re-hospitalization rate will be compared with a contemporary cohort (matched for age, gender, renal replacement therapy and co-morbidities). Our protocol has been approved by research ethics board (UHN: 12-5397-AE). Written informed consent for participation in the study will be obtained from participants.

**Discussion:**

This report serves as a blueprint for the design and implementation of a novel health service delivery model for home dialysis patients. The major goal of the HDVW initiative is to provide appropriate and effective supports to medically complex patients in a targeted window of vulnerability.

**Trial registration:**

(NCT01912001).

## Background

Patients with end-stage renal disease (ESRD) have a high burden of co-morbidity. The most common renal replacement therapy (RRT) in North America is conventional, thrice weekly, in-center hemodialysis (CHD). Home dialysis modalities (peritoneal dialysis (PD) and home hemodialysis (HHD)) are comparably under-utilized, but RRT in the home setting offers greater quality of life [1,2], higher patient satisfaction [3,4] and several other clinical advantages, including better survival compared to CHD [5-10].

These clinical advantages coupled with greater cost-efficacy have made expansion of home dialysis programs a mandate for many healthcare providers [1,11]. Although factors influencing technique failure and recruitment challenges to home dialysis have been examined previously [12-14], the absolute growth of home dialysis remains modest. The underlying reasons for inflow and outflow of patients through home dialysis programs suggest a role for interventions, which may enhance benefits and allay concerns [15]. Conceptually, an innovative care model that provides support to patients would improve retention by decreasing preventable causes of attrition and might also enhance recruitment through improving quality of support of the chronically ill at home.

Periods of transition from acute care to other settings are thought to represent a window of exaggerated patient vulnerability [16,17]. This is because several aspects of the care plan for complex patients may have changed during hospitalization [18,19]. Multiple categories of risk are prevalent during such transitions. These include changes in medication type, dosing and interaction issues, susceptibility to nosocomial infections, confusion regarding follow up, and failure to communicate with primary care providers. This subtlety is not lost on dialysis patients, who rate coordination of care between nephrologists and other physicians lower than other domains of patient satisfaction [3].

Meeting the logistical challenges of improving transitions hinges critically on optimizing clear and timely communication. Thus, care models, which utilize the specialized knowledge of physicians to enhance communication within the healthcare team, are likely to be beneficial. Virtual wards (VW) are a new model of integrated care that seeks to leverage the infrastructure, “systems, staffing and daily routines of a hospital ward…”, to provide services to patients who are not physically admitted to hospital [20]. We have developed a VW model that will capitalize on the infrastructure built for the running of a home dialysis program (PD and HHD). We envisage this VW to function as a pro-active, systematic management tool for vulnerable patients. We hypothesize that implementation of the VW will mitigate gaps in care that occur following care transitions and will facilitate a supported return to baseline health status.

### Objectives

The home dialysis virtual ward will aim to enhance patient care in the following domains:

1. Dialysis prescription and adherence.

2. Morbidity associated with dialysis therapy.

3. Burden associated with travel time for patients.

4. Medication reconciliation.

5. Symptom management.

6. Coordination of care among participating providers.

7. Patient satisfaction.

## Methods/design

### The virtual ward intervention

The home dialysis VW protocol, tools, and evaluation system was developed through an adaptive design process. The members of the VW design team include a physician, pharmacist, dietician, home dialysis nurse, and social worker. Our protocol has been approved by research ethics board (UHN: 12-5397-AE). Written informed consent for participation in the study will be obtained from participants.

The VW intervention consists of a scheduled once to thrice - weekly physician or nurse telephone-administered standardized patient assessment tool. This tool was developed through iterative steps based on input from nephrologists (MS and CC), home dialysis nurses, and renal dieticians. The tool was vetted through dedicated nephrology divisional rounds at the University of Toronto. Feedback from academic nephrologists as well as allied health care staff was incorporated into the final version.

The standardized patient assessment tool serves as a data collection, and clinical form. Relevant domains are identified including: 1. Indication for admission to the VW; 2. Dialysis prescription; 3. Demographic and comorbidity data; 4. Medication reconciliation and 5. Symptom assessment.

The VW nephrologist admits patients and administers the standardized patient assessment tool over the telephone during scheduled VW appointments. Subsequent use of the tool proceeds 1–3 times per week by a member of the team. Patients are admitted to the VW for a planned duration of 14 days and discharged at the discretion of the health team.

### Inclusion criteria: virtual ward patient admission criteria

Consecutive patients under the home dialysis programs (PD and HHD) are admitted to the VW because of anyone of the following:

1. Discharge from hospital following an inpatient admission.

2. Medical procedure (e.g. access procedure).

3. Treatment with antibiotics.

4. Completion of home dialysis training program.

### Exclusion criteria

Patients are excluded from the virtual ward admission if they refuse to provide consent, or are unable to participate (e.g. language barrier).

### Outcome measures

The modified Charlson Comorbidity Index will be used to measure our patients’ case-mix [21]. Additional co-morbidities will also be recorded. The symptom assessment component is based on the validated Modified Edmonton Symptom Assessment Scale [22] and will be supplemented to include the 11 most common symptoms associated with ESRD [23]. The symptom scale also includes a free-form option for patients to report additional symptoms as recommended by Davison [24]. Each home dialysis patient is given a hardcopy symptom reference form. This form includes a visual analog scale that can be referred to at home during administration of the standardized patient assessment tool. This is done to maximize the ease of understanding of the symptom questions and rating scale. All patient materials are designed at a 6^th^ grade level. Aggregate demographic and symptom prevalence data will be presented (Additional file 1).

### Patient satisfaction survey

The Patient satisfaction questionnaire was developed to measure patient opinion regarding this new model of care. The measurement tool was modeled on a similar survey by Juergensen et al. [4], which was fine-tuned using the same iterative process as described for the standardized patient assessment tool. This questionnaire is organized into 4 distinct sections. Section 1 collects demographic information; Section 2 measures the impact of the VW on overall health and likelihood of requiring (re)admission to hospital; Section 3 asks questions about potential ‘coordination of care’ benefits; and, the last section pertains to domains of patients’ personal lives that were thought to be impacted by admission to the virtual ward. A hardcopy questionnaire will be given to all patients upon VW discharge. The questionnaire will be returned anonymously in a postage-paid envelope provided to encourage honest responses.

### Primary outcome

Our primary outcome is to identify the number of care gaps and interventions that the HDVW achieved.

### Secondary outcomes

Our secondary outcomes will include:

1. Within subject differences in symptoms score at baseline and at discharge of VW.

2. Number of re-hospitalizations

3. Rate of re-hospitalization

4. Patient satisfaction

5. Patient encounter time (per VW admission)

6. Stratification of care gaps which are associated with re-hospitalization

7. The VW re-hospitalization rate will be compared to the hospitalization rate of a contemporary cohort (matched for age, gender, renal replacement therapy and co-morbidities).

Admission to the VW will be described in absolute terms and expressed as proportion of total patients admitted per week. Travel time to home dialysis unit and whether the dialysis is performed independently or dependently is ascertained from the patient and/or primary nurse. Dialysis independency is defined as self-reliance on the following three domains: dialysis setup and equipment maintenance, cannulation or catheter access, and dialysis therapy management.

Identification of gaps in care will be assessed by measuring discrepancies between prescribed versus delivered management strategies documented at entry to the VW. Gaps in care will be categorized as 1. Changes to dialysis prescription; 2. Coordination of care; 3. Medication changes; and 4. Symptoms management. The percentage of VW patients with identifiable gaps in care and the types of gaps will be described. We will also stratify the types of care gaps which are associated with re-hospitalization.

Rates of hospital re-admission, un-scheduled home dialysis unit, emergency room, and in-center backup visits will be recorded prospectively for a planned 3-month period and expressed as health-services utilization rates per patient-year. Re-hospitalization rates will be compared before and after the intervention within each home dialysis program.

**Figure 1:**
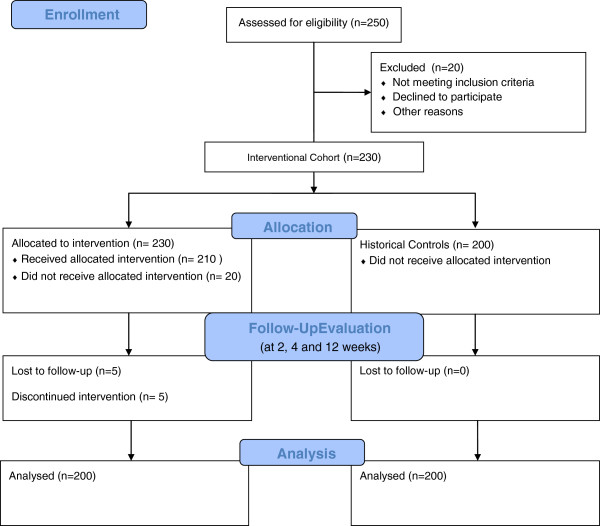
Projected virtual ward study design flow diagram.

VW re-hospitalization rate will be compared to the hospitalization rate of a contemporary incident ESRD patient cohort through an existing collaborative effort by Oliver et al. [25]. The cohorts will be matched for age, gender, renal replacement therapy and co-morbidities (Figure 1).

### Implementation

The HDVW project will be implemented in a Vanguard fashion. A 3 month feasibility phase will be used to enroll 20 consecutive home dialysis patients at the University Health Network. Once the pilot phase of the project is achieved, enrollment from the other sites will begin with a cumulative enrollment target of 200 home dialysis patients during a 9-month period. Our target is based on previous published work in which 400 consecutive general internal medicine patients had 76 adverse events during the 2 weeks following hospital discharge. Of these adverse events, 62% were deemed either preventable or ameliorable [17]. Given that patients with ESRD are more complex than general internal medicine patients, we would estimate conservatively that our cohort would encounter a minimum of 40 events of which the majority may be preventable or ameliorable.

## Discussion

This report describes the design, implementation and study protocol for the HDVW. We believe that the home dialysis patient population is uniquely poised to benefit from this intervention given that our patients are medically complex and commonly receive care from multiple providers. We chose to target our intervention to periods of care transitions, which is an interval of particular vulnerability [16,17]. We hypothesize that gaps in care are common in this setting and aim to develop a systematic description of these issues.

Intensive attention to care gaps in a vulnerable patient population may be predicted to have a number of salutary effects. Two prior randomized studies have shown that multi-disciplinary hospital avoidance schemes can be effective [16,26]. Naylor et al. randomized 363 frail elderly patients to comprehensive discharge planning and home follow-up by gerontology specialist advanced practice nurses (APN) versus standard discharge planning [26]. APNs used joint clinical decision making with physicians in this model. Patients allocated to the APN group had a 16.8% absolute reduction in re-admissions after 24 weeks and consumed approximately $600,000 less in medicare reimbursement charges. Coleman et al. studied high-risk elderly patients [16]. This trial randomized 750 patients to an intervention arm featuring a “transition coach” versus standard discharge planning. Similar to the Naylor study, the transition coach was an APN. The intervention focused on four domains including assistance with medication self-management, use of a patient-owned health record, timely follow-up with physicians, and identifying individualized “red flags”, indicative of a worsening condition. APNs coached patients on these goals before discharge as well as during a home visit and subsequent telephone conversations spanning 28-days post discharge. The adjusted odds ratio of re-admission was 0.64 (95% CI, 0.42-0.99; p = 0.04) at 90 days. The transition coach saved an estimated $300,000 per year by reducing re-admissions.

Improved symptom management has the potential to minimize patient-morbidity and may reduce un-scheduled health-system utilization such as emergency room or walk-in clinic visits. Our intervention specifically targets serial measurement of symptoms score which will allow early identification and optimization of symptoms. Similarly, timely changes in dialysis prescription may decrease the likelihood for extra-cellular fluid overload, which may prevent future hospitalization. Finally, adjustment of medications to reflect evidence-based practice or to minimize risks of drug-interactions is of clear benefit.

While the HDVW may serve to improve patient care, it is equally important to balance the utility of any intervention with its intrusiveness. Patients’ perceptions and satisfaction with the VW service will therefore be analyzed. Patient satisfaction is recognized as an important clinical outcome, which will also provide us an important quality performance measure [27,28].

Implementation of the HDVW strategy will undoubtedly change work flow within the clinical team. It will therefore be important to quantify the amount of time each VW ward admission will require. It is reasonable to speculate that if our present care model is deemed successful, regionalization may ultimately prove to be the most cost efficient approach to broadening availability of this health service offering.

Although the VW model proposed herein does not require additional infrastructure, it is important to recognize potential barriers to its implementation. As a result, we elected to conduct our study in a Vanguard fashion so that a proof of concept feasibility demonstration could be achieved. We envision that our strategy will be cost-neutral, while providing a patient centered sub-specialist delivery service.

In summary, we propose a novel health service delivery model tailored for the home dialysis population. We hypothesize that our medically complex patient population will benefit from our intervention during periods of high vulnerability. We are specifically targeting the effect of the HDVW on gaps of care, patient satisfaction and hospital re-admissions. Given that there may be barriers to the HDVW implementation, we elected to perform a Vanguard design as a proof of concept demonstration. We believe that the results of our study will highlight the care gaps that exist in our patient population. This information will inform a potential re-alignment of our resources to shift from a hospital-centric to a more patient-centered care delivery model.

## Competing interests

The authors declare that they have no competing interests. MJ Oliver is the co-creator of the Dialysis Measurement Analysis and Reporting System.

## Authors’ contributions

All authors contributed important intellectual content and participated in critical revisions to the manuscript. All authors read and approved the final manuscript.

## Pre-publication history

The pre-publication history for this paper can be accessed here:

http://www.biomedcentral.com/1471-2369/15/33/prepub

## Supplementary Material

Additional file 1 **Home Dialysis Patients Symptom Reference Form**^**1**^**.** Click here for file
