# Supplementary material for: Rationale for a home dialysis virtual ward: design and implementation
Source: BMC Nephrol. 2014 Feb 14;15:33. doi: 10.1186/1471-2369-15-33 (PMC3930556; doi:10.1186/1471-2369-15-33)
Supplement: Additional file 1 — Home Dialysis Patients Symptom Reference Form 1 . [file 1471-2369-15-33-S1.pdf]

Home Dialysis Patients Symptom Reference Form[1]

How bad are your symptoms of kidney disease?

Please look at the picture below to help you tell us about your symptoms.

You may choose a score of zero, which means “no symptoms”, or a score of 10, which means “very bad symptoms”. Otherwise, choose a point somewhere on the line between 0 to 10, which is equal to the severity of your symptoms.

	**No Symptoms**										**Very Bad Symptoms**
1. Shortness of breath	0	1	2	3	4	5	6	7	8	9	10
2. Cough	0	1	2	3	4	5	6	7	8	9	10
3. Fatigue/tiredness	0	1	2	3	4	5	6	7	8	9	10
4. Lack of appetite	0	1	2	3	4	5	6	7	8	9	10
5. Nausea	0	1	2	3	4	5	6	7	8	9	10
6. Vomiting	0	1	2	3	4	5	6	7	8	9	10
7. Diarrhea	0	1	2	3	4	5	6	7	8	9	10
8. Constipation	0	1	2	3	4	5	6	7	8	9	10
9. Pain	0	1	2	3	4	5	6	7	8	9	10
10. Itching	0	1	2	3	4	5	6	7	8	9	10
11. Other:_______________	0	1	2	3	4	5	6	7	8	9	10
	**No Symptoms**										**Very Bad Symptoms**

References:
[1] Murtagh FEM, Addington---Hall J, and Higginson IJ. The Prevalence of Symptoms in End---Stage Renal Disease: A Systematic Review. Advances in Chronic Kidney Disease 2007;14:82---99.
